# Stakeholder views of ethical guidance regarding prevention and care in HIV vaccine trials

**DOI:** 10.1186/1472-6939-15-51

**Published:** 2014-06-30

**Authors:** Rika Moorhouse, Catherine Slack, Michael Quayle, Zaynab Essack, Graham Lindegger

**Affiliations:** 1 Faculty of Health Sciences, Simon Fraser University, 8888 University Drive, Burnaby, British Columbia, Canada; 2 HIV AIDS Vaccines Ethics Group, School of Applied Human Sciences, College of Humanities, University of KwaZulu-Natal, Pietermaritzburg, Private Bag X01, Scottsville, South Africa

## Abstract

**Background:**

South Africa is a major hub of HIV prevention trials, with plans for a licensure trial to start in 2015. The appropriate standards of care and of prevention in HIV vaccine trials are complex and debated issues and ethical guidelines offer some direction. However, there has been limited empirical exploration of South African stakeholders’ perspectives on ethical guidance related to prevention and care in HIV vaccine trials.

**Methods:**

Site staff, Community Advisory Board members and Research Ethics Committee members involved with current HIV vaccine trials in South Africa were invited to participate in an exploration of their views. A questionnaire listed 10 care and 10 prevention recommendations drawn from two widely available sets of ethical guidelines for biomedical HIV prevention trials. Respondents (n = 98) rated each recommendation on five dimensions: “Familiarity with”, “Ease of Understanding”, “Ease of Implementing”, “Perceived Protection”, and “Agreement with” each ethical recommendation. The ratings were used to describe stakeholder perspectives on dimensions for each recommendation. Dimension ratings were averaged across the five dimensions and used as an indication of overall merit for each recommendation. Differences were explored across dimensions, between care-oriented and prevention-oriented recommendations, and between stakeholder groups.

**Results:**

Both care and prevention recommendations were rated highly overall, with median ratings well above the scale midpoint. In general, informed consent recommendations were most positively rated. Care-related recommendations were rated significantly more positively than prevention-related recommendations, with the five lowest-rated recommendations being prevention-related. The most problematic dimension across all recommendations was “Ease of Implementing,” and the least problematic was “Agreement with,” suggesting the most pressing stakeholder concerns are practical rather than theoretical; that is, respondents agree with but see barriers to the attainment of these recommendations.

**Conclusions:**

We propose that prevention recommendations be prioritized for refinement, especially those assigned bottom-ranking scores for “Ease of Implementing”, and/ or “Ease of Understanding” in order to assist vaccine stakeholders to better comprehend and implement these recommendations. Further qualitative research could also assist to better understand nuances in stakeholder reservations about implementing such recommendations.

## Background

South Africa has identified the development of safe and effective HIV vaccines as a health priority [1,2]. The country has developed national standards on ethics for HIV vaccine research, namely the Medical Research Council of South Africa’s *Guidelines on Ethics for Medical Research: HIV preventive vaccine trials*[1], and domestic vaccine stakeholders are also governed by international guidelines on ethics for HIV prevention trials [3,4]. To-date however there has been limited empirical exploration on whether current normative guidance is perceived to offer sound direction about current ethical complexities in HIV vaccine trials (HVTs).

In 2011 there were 5.6 million South Africans living with HIV [5]. While South Africa now runs a large antiretroviral therapy (ART) treatment program, the number of ART-eligible South Africans continues to exceed coverage [6]. Research into new prevention technologies and methods is vitally important in the battle against the epidemic. To this end, South Africa has conducted numerous HVTs since 2003. There are five sites currently implementing preventive HVT protocols, however, a massive scale up of site capacity is expected as South Africa prepares to implement a phase III licensure trial in the country in approximately 2015.

In South Africa and other host countries for HVTs, participants are often drawn from communities with relatively poor socio-economic conditions and strained health-care resources yet funded by agencies from resourced nations [7,8]. These imbalances frame intense ethical debate regarding sponsor-investigator obligations vis-à-vis the rights and wellbeing of participants and participating communities. Two related and divisive ethical debates in HVTs are the responsibilities of sponsor-investigators to offer a high standard of HIV prevention and to take steps to address the medical needs identified in trials, including HIV.

Individuals are deemed eligible for late-phase HVTs because they are at high risk of HIV infection. Many commentators have deliberated on the responsibilities of sponsor-investigators to ensure that such participants receive access to effective methods to prevent HIV acquisition and how such decisions should be made, particularly where such methods are not routinely available [9-12]. Considerations include defining when scientific thresholds have been met for ‘proving’ the effectiveness of prevention methods [13], obtaining the ‘approval’ of relevant authorities for such methods, and getting stakeholder agreement which may require overcoming possible cultural objections to these prevention methods [14]. Some debated concerns include the possible biological and behavioural interactions between prevention methods [15], determining how much risk reduction methods will decrease expected sero-incidence rates and affect study power [10,16-18], the impact on product licensure [15,19] and the role tension between promoting risk-reduction and promoting scientific objectives [3].

Some participants in HVTs will acquire HIV infection despite access to a range of effective HIV prevention methods. In addition, other health-care problems are likely to be identified by site staff [20,21]. There has been intense discussion about the obligations of sponsor-investigators to address the HIV-related needs of participants, particularly in settings where access to comprehensive care and ART is not reliably available [22-24]. The discussion has also expanded to include actions researchers should take to address medical problems apart from HIV infection, and obligations to build local capacity for health care [25,26].

Debates have centred on the ethical justification underpinning the responsibility to address participants' medical problems by sponsor-investigators [23,24] and identifying reasonable limits on such responsibilities [27]. Some commentators maintain that a high standard of care for HVT participants may introduce unacceptable local inequalities or constitute an ‘undue inducement’ [28]. General ethical guidelines do not provide complete answers to these complex questions surrounding prevention and care in HVTs. However, international documents do offer certain recommendations. Trial stakeholders in South Africa can also turn to domestic guidance adapted from the UNAIDS (2000) guidelines [1].

While defining ethical norms in HIV prevention research is complex, determining the process by which ethical standards are to be established can be an equal challenge. One such complexity is the role of consultation in guideline-development. When UNAIDS released its *Ethical Considerations in HIV Preventive Vaccine Research* guidelines (2000) [29] some commentators argued the guidelines did not represent international agreement; but rather reflected the opinions of the small group of drafters [30]. This contention highlights stakeholder interest in just and participatory methods for determining guideline content.

Other complexities include whether guidelines should be aspirational or mandatory. Should they represent ideals to strive for without threat of retribution (so called aspirational guidance) or rather represent a basis for sanction (mandatory guidance) [31]? Should guidelines present a substantive position or a decision-making standard for resolution of divisive issues [7]? In addition, it has been noted that a guideline’s formal purpose (e.g. aspirational, regulatory, or educational) may or may not correspond with the intended audience’s reading of the guideline [32]. Commentators have noted that guidelines can be extolled *and* ‘exhibited in practice’ (functional norms) or extolled *but* ‘ignored’ (non-functional norms) [33]. If guidelines are ignored, there are conceivably various reasons for that: there could be low awareness of the recommendation; it could be phrased poorly or ambiguously; or it could be considered unachievable or irrelevant to local context by the intended audience. Learning more about stakeholder perspectives of ethical guidelines might, therefore, assist to improve their status as functional norms. The latter observation intersects with a broader debate about the role of empirical data in bioethics [34-37]. Sugarman and Sulmasy [38] propose several roles for empirical research in bioethics including describing facts relevant to normative arguments. As set out in Slack [39], empirical research can inform a critical reflection on ethical norms [34] by, for example, shedding light on ethical problems that require attention [40] or by providing the details that inform more responsive ethical recommendations [41]. This empirical study describes stakeholder perspectives regarding key guidance to aid in creating guidance that is more responsive to these perspectives and to the implied needs of guidance users.

While debate continues on the appropriate relationship between empirical and conceptual modes of inquiry, there seems to be agreement that empirical findings can and do contribute to the discourse on normative ethics. In the Global South, there has been some empirical research focused on the ethical perspectives of research stakeholders [28,42-44] and explorations of perceived challenges by HVT stakeholders in South Africa [45,46]. The UNAIDS-AVAC *Good Participatory Practice Guidelines for Biomedical HIV Prevention Trials* guidelines (2007) (*GPP guidelines)*[4] have been evaluated to establish stakeholder awareness and utilization [47] and to gauge stakeholder perceptions of their relevance [48]. Additionally, empirical explorations of care and prevention *practices* in HIV prevention trials have been undertaken but did not specifically focus on perceptions of guidance [21,49,50]. We undertook a descriptive ethics study to fill this gap and focus on two sets of international ethical guidelines: *Ethical considerations in biomedical HIV prevention trials* (2007) *(UNAIDS guidelines)*[3] and the *GPP guidelines*[4].

The *UNAIDS guidelines* are framed as ‘suggested guidance’ and the *GPP guidelines* are intended to act as systematic guidance regarding engagement in trials. Some commentators have discussed the possibility that UNAIDS guidance documents set an unpragmatic standard for research stakeholders [28,31]. Although the *UNAIDS guidelines* and recent revisions to both booklets [51,52] have been available for some time, there has been little empirical exploration of the guidance, including whether stakeholders are aware of their recommendations or whether they think the recommendations offer helpful direction on key concerns.

### Study aims

This study aimed to explore South African stakeholder perspectives of ethical recommendations for the complex and controversial issues of prevention and care in HVTs. We sought to identify where ‘functionality’ of these ethical recommendations could be improved. Vaccine stakeholders are the primary audience for our study, including guideline-developers. As such, we conclude with suggested reforms to improve the guidelines and make them more responsive to stakeholder perceptions about their usefulness. The study’s findings, however, may also be of interest to a broader readership concerned with the role of empirical data in contributing to the refinement of ethical norms and recommendations.

The three questions driving this specific study were: 1) which overall ethical recommendations are perceived most and least favourably (on the dimensions of familiarity with, ease of understanding, ease of implementing, perceived protection, and agreement with); 2) whether stakeholders perceive care-related recommendations to be more or less challenging than prevention-related recommendations; and 3) whether differences exist between stakeholder groups regarding their views on ethical recommendations. This study was part of a larger qualitative exploration of care and prevention practices of HVT stakeholders, funded by the Wellcome Trust’s Biomedical Ethics Program.

## Methods

Our exploratory inquiry used a quantitative survey design, as this design is well suited to describing knowledge, beliefs and attitudes [38]. The survey allowed us to sample a large number of respondents within a relatively short data collection period and to make comparisons across stakeholder groups, which was a priority for analysis. There were a limited number of open-ended items included in the questionnaire, however, in the interests of space this data is not reported here.

### Instrument development

The study team identified 20 key ethical recommendations from the *UNAIDS guidelines* and *GPP guidelines* that provided direction regarding prevention and care services that should be made available to participants in HVT, how such services should be ensured, or how decisions on care and prevention should be made. Recommendations were selected for inclusion in the questionnaire if they were relevant to the complex, unresolved and topical care and prevention issues being debated in ethics discourse [15,19,27,50].

A questionnaire was developed to allow respondents to evaluate each ethical recommendation with Likert-type ratings on various dimensions (see Additional file 1). These dimensions were “Familiarity with”, “Ease of Understanding”, “Ease of Implementing”, “Perceived Protection”, and “Agreement with” the recommendation. Averaged ratings across these dimensions for each recommendation were taken as scales indicating the recommendation's overall perceived merit. Summaries for each dimension and overall merit were calculated to compare overall perceptions of care- and prevention-oriented recommendations.

The questionnaire was piloted with a small ethically knowledgeable sample (n = 20). Sixteen individuals reviewed the items (for relevance to study objectives, clarity, and questionnaire structure) and eleven completed the questionnaire. Of these pilot respondents, seven both reviewed the items and completed the questionnaire. Poor items were revised or discarded when preliminary item analysis indicated they were too ambiguous or otherwise unsuitable for inclusion. All materials were developed in English and translated on request.

### Sampling

Three key stakeholder groups were selected for participation: current trial site-staff involved in HVT implementation at five sites conducting HVTs, current members on Community Advisory Boards (CABs) at the same sites, and Research Ethics Committee (REC) members on RECs known to have reviewed current HVTs in South Africa.

The Principal Investigators at each HVT site agreed to sensitization meetings with site-staff and CABs to discuss participation and, with their approval, provided full lists of eligible site-staff and CAB members for sampling. Chairs of eligible RECs were approached via email to negotiate access and all were willing to provide full membership lists except one REC, which sent a shorter list of members willing to be approached. Since the pool of eligible participants was small, sampling was exhaustive rather than random.

### Data collection

Data collection was conducted between June 2010 and July 2011. Standard Operating Procedures were developed for the collection of questionnaire data, anonymisation, documentation, and data integrity and security. When stakeholders first responded, an information sheet including an informed consent form was provided digitally. Typically trial site staff and RECs responded by email, while CAB members participated at a CAB meeting. Participants received a modest reimbursement for their time and inconvenience, approved by local RECs.

### Data analysis

The Likert-type dimension ratings for the list of ethical recommendations were designed to form multi-item measures as follows: (1) Summary scales representing perceived overall merit of *each* ethical recommendation, (2) Summary scales representing perceived overall merit for care recommendations combined and prevention recommendations *combined*, and (3) Summary scales for each *dimension* across care recommendations combined and across prevention recommendations combined. In each case reliability was assessed with Cronbach’s Alpha to justify scaling items along these lines. Summary scale scores were tabulated and interpreted as more or less ‘problematic’ or having more or less ‘merit’ depending on their relative ranking. These evaluations did not challenge the absolute merit of the recommendations; rather, a more ‘problematic’ recommendation was identified as one where its perceived merit was lower *relative to other recommendations*.

Scores were calculated as follows: (1) Since scores for each dimension of a single recommendation ranged from 1 to 5, a *global merit score* for each recommendation was added up by combining the five individual dimension scores for that recommendation. Each recommendation thereby received a single summated score ranging from a possible 5 to 25; (2) Since the overall merit score for each ethical recommendation ranged from a possible 5 to 25, overall merit scores for the 10 care recommendations combined added up to a summated score ranging from a possible 50 to 250; the same summation was done for the 10 prevention recommendations combined; (3) Scores for each dimension of every individual recommendation were then summated again; instead of adding dimension scores together within a single recommendation, this summation added up all individual “Familiarity with” dimension items for care recommendations. As there were ten care recommendations, there were ten scores that were summated into one combined score for care recommendations ranging from a possible 10 to 50 for the “Familiarity with” dimension. The same process was repeated for the other dimensions, yielding five combined dimension scores for care recommendations. The same five combined dimension scores were calculated for prevention recommendations.

For (1), *perceived global merit of each ethical recommendation*, a z-score was calculated for the merit rating of each ethical recommendation to more accurately compare scales with different levels of variability. This was particularly important because ratings of some recommendations had very little variability while others exhibited a great deal. These standardized scores for recommendations were then ranked from 1 (the highest scoring recommendation) to 20 (the lowest scoring recommendation). A z-score was also calculated and ranked for each recommendation's five individual dimensions. Since there were 20 recommendations that were each scored on five dimensions, there were 100 scores in total from 1 (the highest scoring recommendation-dimension score) to 100 (the lowest scoring recommendation-dimension score).

For (2) and (3), *global and dimension-specific merit of care recommendations combined versus prevention recommendations combined,* paired t-tests were used to determine whether differences in median scores were significant. Median and interquartile range (IQR) were determined for responses ‘overall’ and by stakeholder group. A Kruskal-Wallis (KW) test was conducted to explore differences in dimension-specific perceptions of care versus prevention recommendations across stakeholder groups. The KW test was selected because it is non-parametric and does not require that sample data are even or distributed normally, and we anticipated small uneven sample sizes given the small uneven sized populations from which the study would sample. To offset family-wise error, a conservative Bonferoni correction set alpha at 0.003 for paired t-tests and KW tests. All quantitative analyses were performed in Statistical Analysis System software.

### Ethical considerations

Ethics approval was obtained for this study from the University of Kwazulu-Natal Biomedical Research Ethics Committee (BE 241/09) and from Simon Fraser University's Research Ethics Board. Ethical approval was also obtained from all Research Ethics Committees with jurisdiction over the HVT sites at which data was collected.

## Results

A total of 98 questionnaires were completed (69 from CABs, 21 from RECs, and 8 from site staff) with a response rate of 43%. Internal consistency for global merit scores was >0.6 in all cases for each recommendation statement, when recommendations were summarized by category (i.e. prevention recommendations combined and care recommendations combined), and for the global merit scores for care and prevention combined (i.e. combining “Familiarity with”, “Ease of Understanding”, “Ease of Implementing”, “Perceived Protection”, and “Agreement with” across all care recommendations and the same across all prevention recommendations). This item analysis indicates that all scales had sufficient reliability for the purposes of this study.

### Ethical recommendations

**Figure 1:**
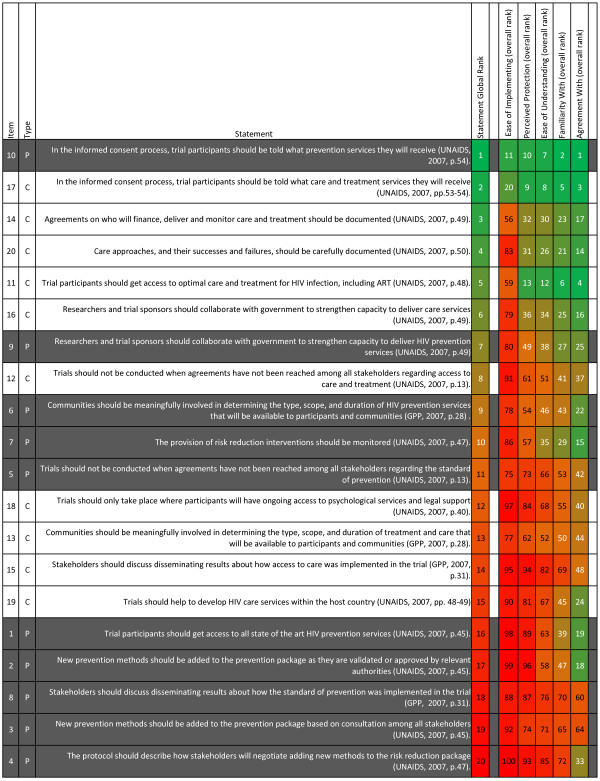
**Recommendations ranked from top to bottom scores and dimensions ranked from top to bottom scores.** This table shows the ethical recommendations in order from the most positive global rating (1) to the least positive global rating (20). Each ethical recommendation was rated by participants on “Ease of Implementing”, “Perceived Protection”, “Ease of Understanding”, “Familiarity with”, and “Agreement with”. For each ethical recommendation, we worked out the average score for each dimension. Since there were 20 statements that were each ranked on five dimensions, there were 100 scores in total. We ranked these in order from 1 (the highest scoring recommendation-dimension score) to 100 (the lowest scoring recommendation-dimension score). We used green shading to highlight the highest rankings, orange to represent the middle-rankings, and red to highlight the lowest rankings.

The first question driving this study was *which overall ethical recommendations are perceived as having more or less merit* (or, which recommendations are perceived as more or less challenging by stakeholders)? Figure 1 presents all ethical recommendations in order from top overall merit rank to bottom overall merit rank. The dimension scores alongside were ranked from 1 to 100 across dimensions to identify the most problematic and least problematic recommendations and dimensions.

All median scores were reasonably high on the overall merit scale, ranging from 18 out of 25 (recommendation #4) to 25 out of 25 (recommendation #10) (tabulated scores not shown). The majority of top-ranked ethical recommendations on overall merit were care-related, including, *Trial participants should get access to optimal care and treatment for HIV infection, including ART* (recommendation #11). The majority of bottom-ranked ethical recommendations on overall merit were prevention-related, including, *Trial participants should get access to all state of the art HIV prevention services* (recommendation #1). The highest ranked recommendations on overall merit were both informed consent recommendations, namely recommendation #10, *In the informed consent process, trial participants should be told what prevention services they will receive*, and recommendation #17, *In the informed consent process, trial participants should be told what care and treatment services they will receive*. The five lowest ranked recommendations were all prevention-related: *The protocol should describe how stakeholders will negotiate adding new methods to the risk reduction package* (recommendation #4), *New prevention methods should be added to the prevention package based on consultation among all stakeholders* (recommendation #3)*, Stakeholders should discuss disseminating results about how the standard of prevention was implemented in the trial* (recommendation #8), *New prevention methods should be added to the prevention package as they are validated or approved by relevant authorities* (recommendation #2), and *Trial participants should get access to all state of the art HIV prevention services* (recommendation #1).

### Dimension scores for ethical recommendations

The five dimension scores for each ethical recommendation were also ranked and these rankings are presented in Figure 1. Overwhelmingly, the dimension that ranked lowest across recommendations was “Ease of Implementing”. Indeed, for every single recommendation, “Ease of implementing” was the lowest ranked of the five dimension scores. Conversely, the dimension that ranked highest across recommendations was “Agreement with”. For all five most challenging recommendations, scores for the “Agreement with” dimension had higher ranks than the other four dimensions. In fact, “Agreement with” for recommendation #2 and recommendation #1 were in the top quarter of 100 possible dimension rankings despite the problematic ratings on other dimensions for these recommendations.

The bottom-ranked recommendation under each dimension was identified to clarify which recommendations were most problematic in each dimension. The recommendation that was most problematic in terms of “Familiarity with”, “Ease of Implementing”, and “Perceived Protection” was recommendation #4, *The protocol should describe how stakeholders will negotiate adding new methods to the risk reduction package.* Most problematic in terms of “Ease of Understanding”, was recommendation #2, *New prevention methods should be added to the prevention package as they are validated or approved by relevant authorities* and most problematic for “Agreement with” was recommendation #3, *New prevention methods should be added to the prevention package based on consultation among all stakeholders*.

### Prevention versus care recommendations

The second question driving this study was, *do stakeholders perceive care recommendations to be more or less challenging than prevention recommendations?* Care recommendations were scored higher overall than prevention recommendations. (p = 0.002). Care recommendations were also scored higher for the dimensions “Familiarity with” (p = 0.004), “Ease of Understanding” (p = 0.010), “Perceived Protection” (p = 0.969) and “Ease of Implementing” (p < 0.001). After adjusting for multiple testing, differences in median score between care and prevention recommendations were considered significant (p = 0.003) overall, and for the dimensions “Ease of Implementing” and “Perceived Protection”.

The study’s third question was w*hat differences exist between stakeholder groups (if any) regarding their views on ethical recommendations?* For prevention recommendations there was a significant difference in views among the three stakeholder groups for the “Ease of Implementing” (p < 0.001) and “Perceived Protection” (p < 0.001) dimensions. For care recommendations there was also a significant difference in views between the three stakeholder groups for the “Ease of Implementing” (p < 0.001) and “Perceived Protection” (p = 0.002). The REC group assigned noticeably lower scores across recommendations and dimensions when compared to the other two stakeholder groups.

## Discussion

### Overall merit of ethical recommendations

The findings indicate that both care and prevention recommendations were rated favourably overall. This result reflects well on the international guidelines as it suggests that these recommendations were viewed positively by stakeholders operating at the coal-face of HVTs.

The prominence of informed consent recommendations is not surprising. The high profile of consent requirements in ethics discourse generally [53] and the presence of well accepted mechanisms to implement consent requirements (such as written forms) might explain the especially high rankings for “Familiarity with” and “Ease of Understanding” for recommendations that participants be informed about the care or prevention efforts they will receive. High level of agreement with these two informed consent recommendations is consistent with the wide recognition and popular acceptance of the principle of respect for autonomy [54-56] and suggests that these stakeholders agree that participants should be aware of the package on offer as part of their overall participation [39,57]. A previous study also found that HVT stakeholders in South Africa spontaneously identified 'informed consent' most frequently as a critical issue for HVTs [46].

One of the highest-ranked ethical recommendations was *'Trial participants should get access to optimal care and treatment for HIV infection, including ART*' (recommendation #11). The high dimension rank for “Perceived Protection” (6/100) by this recommendation suggests that South African stakeholders are fairly unified in the view that high quality HIV care protects participant rights and promotes their welfare. It is not clear whether respondents index ‘optimal’ to international or national standards, but appear to endorse access to an antiretroviral regimen as a core protection. “Familiarity with” ranked high for this recommendation (4/100) which is explained by the high-profile debate about ART-access in such trials [7,23,58].

The findings show that most top ranked recommendations on overall merit were care-related and bottom ranked recommendations were prevention-related. As alluded to above, higher scores assigned to care recommendations might be explained by years of deliberation - and emergent consensus, as some claim [59] about sponsor-investigators' responsibilities to ensure access to ART. Increased access to such treatment facilitated by national governmental programs as well as donor-funded initiatives [60] and the emergence of collaborations to meet such responsibilities could also explain the higher scores assigned to care recommendations. Conversely, participant access to effective preventive methods are likely to reflect a more recent, contemporary and pressing controversy for stakeholders, given the latest successes of prevention products in trials such as PrEP [61] and microbicides [62]. While the issue of adding new methods to prevention packages did feature in early papers on HVTs [7] it is recent successes in biomedical prevention trials that have brought this debate to prominence [19]. There is arguably less consensus on how recent results should be translated into standards of prevention in trials than there is for access to ART.

### Care versus prevention recommendations

A close read of the dimension scores indicated that low scores assigned to “Ease of Implementing” contributed substantially to the pattern of lower ranking for prevention recommendations. It is worth noting that “Agreement with” was ordered high in the dimension rankings with each of the bottom 5 recommendations; and “Agreement with” was even positioned decently when ranked against all other dimensions for all recommendations. This finding suggests that respondents agreed with these recommendations- all five of which are prevention-related- but viewed them as difficult to apply in practice, suggesting that the solution may be to provide more help in how to realize the standards in practice. This finding intersects somewhat with the observation that merely because an ethical standard is difficult to attain does not render the standard wrong [63].

One possible explanation for low ranking on “Ease of Implementing” and low to moderate ranking on “Ease of Understanding” on prevention recommendations is that respondents may struggle to operationalise broad concepts contained therein, such as ‘*validation*’ (recommendation #2), ‘*approval by relevant authorities*’ (recommendation #2), and *'state of the art*' (recommendation #1). It suggests that stakeholders need more refined or elaborated guidance to help them to implement these recommendations or to realize them in practice, which is a point we will return to under ‘conclusions and recommendations’.

The 3 bottom-ranked recommendations out of 20 are all concerned with inter-stakeholder engagement activities regarding prevention, therefore it is possible that respondents may anticipate tensions between stakeholders regarding standards of prevention when trying to 'negotiate' (recommendation #4), 'consult' (recommendation #3), and 'discuss' (recommendation #8). This conclusion is supported by significant inter-stakeholder differences (see below).

### Most problematic recommendations by dimensions

Recommendation #4 [*The protocol should describe how stakeholders will negotiate adding new methods to the risk reduction package*] was the lowest recommendation for the following dimensions - “Familiarity with”, “Perceived Protection” and “Ease of Implementing”. The contribution of these bottom dimension scores to recommendation #4’s overall bottom rank (out of 20) suggests it requires more careful thought and reformulation. It is not clear whether these stakeholders were questioning the overall approach inherent in ‘moral negotiation’ about prevention-related benefits as set out by ethical commentators [64] or whether they were questioning the value of committing to paper plans in this regard because of reservations about preserving flexible innovative responses to participants' prevention needs [57].

Recommendation #2 [*New prevention methods should be added to the prevention package as they are validated or approved by relevant authorities*] was the bottom recommendation under “Ease of Understanding”. As mentioned earlier, more substantive elaboration in the ethical guidance might help stakeholders to understand key concepts such as ‘validation’ (e.g. what threshold of evidence?) and ‘approval’ (e.g. which agencies?) which is a point we will return to under ‘conclusions and recommendations’.

Recommendation #3 [*New prevention methods should be added to the prevention package based on consultation among all stakeholders]* was the bottom-ranked recommendation under “Agreement with” *(64/100)* even while “Agreement with” was a high-ranked dimension generally. While respondents did not disagree, they did not indicate overwhelming support for this recommendation. This indicates that these stakeholders may not share the view that *all* stakeholders should be consulted about this issue, reflecting a possible skepticism about throwing the net too wide for consultation which might delay a timely decision.

### Stakeholder differences

Significant differences were found between stakeholder groups on “Ease of Implementing” and “Perceived Protection” for both prevention recommendations combined and care recommendations combined, with significantly lower scores for the REC stakeholder group. This suggests that specific stakeholder groups hold different views about whether certain recommendations can protect trial participants in the first place, or whether recommendations could ever be implemented in practice. This finding adds a layer of detail to the long-recognized problem that stakeholder consensus about critical components of trials is difficult to achieve [13] and provides some clues about why inter-stakeholder forums to debate such concerns may break down or be marred by disagreement. Prior reflection on difficulties in achieving consensus have centered on inadequate negotiating power held by community stakeholders [65]. This finding may be an important topic for future research because current ethical guidance recommends collective decision- making across stakeholder groups [51] prior to trial commencement regarding prevention and care.

### Limitations

It was a challenge to achieve a representative sample. Response rates were low, especially within the site-staff group (11%). The sampling methods needed to access the target stakeholder groups eliminated the possibility of random sampling, and instead participants self-selected in response to questionnaire outreach. Given the small population sizes, small samples and non-random sampling, the perspectives of eligible stakeholders not included in the sample remain unknown, and may differ from those of study respondents. The study was also regionally specific. Only South African HVT stakeholders were sampled. For these reasons, inference to a larger population of stakeholders is not made from these findings.

Certain stakeholder groups for HVTs were not sampled for this study: sponsors, regulators and HVT participants. Sponsors and regulators were excluded due to the anticipated small numbers; the study team was interested in inter-stakeholder analysis and anticipated not being able to recruit the sample size required to compare such perspectives to other groups. HVT participants were not recruited because of the possible impact on ongoing trials, and because of anticipated burden in securing permissions necessary to access participants. However, further research on HVT stakeholder perspectives would benefit from inclusion of these stakeholder groups.

More broadly, this study adopted an empirical approach to exploring the adequacy of ethical recommendations, whereas some might argue that conceptual analysis is a better approach. However, empirically describing the views of vaccine stakeholders about the merit of various recommendations arguably provides a platform from which to consider how to make guidance potentially more helpful and implementable.

During the study, the *GPP guidelines* (2007) were superseded by a new edition [51], which excluded the recommendations listed as #6 and #8, however the others are still relevant. In 2012, a new point was added to the *UNAIDS guidelines* on Participants who use Intravenous Drugs resulting in a re-release [52] which does not affect the relevance of these questionnaire items.

## Conclusions and recommendations

All recommendations related to prevention and care were rated favourably by our respondents in terms of overall merit which indicated that they are perceived as helpful by core role-players. Care recommendations were rated more favourably than prevention recommendations, therefore we propose that prevention recommendations be prioritized for refinement by guideline drafters, especially those assigned bottom-ranking scores for “Ease of Implementing”, and/or “Ease of Understanding” in order to assist vaccine stakeholders to better comprehend and implement these recommendations.

Based on findings about which dimensions drive lower scores assigned to prevention recommendations, we recommend some elaboration in guidance of the broad concepts to enable stakeholders to better understand and implement prevention recommendations. Such broad concepts include ‘validation’, ‘consultation of all stakeholders’ and ‘approval by authorities’. It is likely that vaccine stakeholders will need more operational guidance on specific issues such as establishing the safety (including combination effects) of the prevention modality for the trial population, establishing clinical benefit of the prevention modality for the trial population [66], determining which role-players in the national context are to be selected for consultation, which activities constitute appropriate ‘consultation’, and which consultative formats (e.g. meetings, protocol reviews) are appropriate. Further qualitative research could also assist to better understand nuances in stakeholder reservations about implementing such recommendations for HVTs, which could be used to better translate broad recommendations into effective practice.

## Competing interests

The authors declare no competing interests.

## Authors’ contributions

CS, MQ, GCL and ZE participated in the design of the study. RM, CS and ZE conducted data collection. RM, CS, MQ, and ZE participated in data analysis, with the assistance of a statistician. RM drafted the original manuscript, with substantive contributions to the final manuscript by CS, MQ and ZE. MQ produced visual representation of the data. All authors read and approved the final manuscript.

## Pre-publication history

The pre-publication history for this paper can be accessed here:

http://www.biomedcentral.com/1472-6939/15/51/prepub

## Supplementary Material

Additional file 1 Study questionnaire for “Stakeholder views of ethical guidance regarding prevention and care in HIV vaccine trials”. Click here for file
