# Supplementary material for: Stakeholder views of ethical guidance regarding prevention and care in HIV vaccine trials
Source: BMC Med Ethics. 2014 Jun 30;15:51. doi: 10.1186/1472-6939-15-51 (PMC4104735; doi:10.1186/1472-6939-15-51)
Supplement: Additional file 1 — Study questionnaire for “Stakeholder views of ethical guidance regarding prevention and care in HIV vaccine trials”. [file 1472-6939-15-51-S1.pdf]

Questionnaire on UNAIDS guidelines

Hello. The following questionnaire will ask you about ethical standards extracted from UNAIDS-WHO (2007) *Ethical considerations in biomedical HIV prevention trials* and UNAIDS-AVAC (2007) *Good participatory practice guidelines for biomedical HIV prevention trials*. The first 10 standards are about prevention and the rest are about care.

Before you begin, please indicate the stakeholder group to which you belong by marking with an X

Site staff member	☐	
CAB member	☐	
REC member	☐	
Regulatory member	☐	
Sponsor	☐	
Other (please specify)	☐	

You may worry that we are testing how much you know about ethical standards. This is not the case. Rather, we hope to identify those areas where ethical standards may have to be clarified, disseminated better, or even changed.

For each ethical standard you will be asked to rate five things:

- How familiar you are with the ethical standard
- How easy you think it is to understand the ethical standard
- How easy you think it is or will be to implement the ethical standard
- How much the ethical standard protects participants
- How strongly you agree with the ethical standard

The relevant ethical standards are written in the left-hand column.

Next to each ethical standard are five rating boxes. Please carefully read the ethical standard and then choose a number between 1 and 5 for each of the five ratings before moving on to the next ethical standard.

For example:

Trial participants should be treated with respect.	☒	5	I am very familiar with this ethical standard	☐	5	This ethical standard is very easy to understand	☐	5	This ethical standard is very easy to implement	☐	5	This ethical standard greatly protects trial participants	☐	5	I strongly agree with this ethical standard
	☐	4		☐	4		☐	4		☒	4		☒	4	
	☐	3		☐	3		☒	3		☐	3		☐	3	
	☐	2	I am not at all familiar with this ethical standard	☐	2	This ethical standard is very hard to understand	☐	2	This ethical standard is very hard to implement	☐	2	This ethical standard minimally protects trial participants	☐	2	I strongly disagree with this ethical standard
	☐	1		☒	1		☐	1		☐	1		☐	1	

1. Trial participants should get access to all state of the art HIV prevention services.	☐ 5 I am very familiar with this ethical standard<br>☐ 4<br>☐ 3<br>☐ 2<br>☐ 1 I am not at all familiar with this ethical standard	☐ 5 This ethical standard is very easy to understand<br>☐ 4<br>☐ 3<br>☐ 2<br>☐ 1 This ethical standard is very hard to understand	☐ 5 This ethical standard is very easy to implement<br>☐ 4<br>☐ 3<br>☐ 2<br>☐ 1 This ethical standard is very hard to implement	☐ 5 This ethical standard greatly protects trial participants<br>☐ 4<br>☐ 3<br>☐ 2<br>☐ 1 This ethical standard minimally protects trial participants	☐ 5 I strongly agree with this ethical standard<br>☐ 4<br>☐ 3<br>☐ 2<br>☐ 1 I strongly disagree with this ethical standard
2. New prevention methods should be added to the prevention package as they are validated or approved by relevant authorities.	☐ 5 I am very familiar with this ethical standard<br>☐ 4<br>☐ 3<br>☐ 2<br>☐ 1 I am not at all familiar with this ethical standard	☐ 5 This ethical standard is very easy to understand<br>☐ 4<br>☐ 3<br>☐ 2<br>☐ 1 This ethical standard is very hard to understand	☐ 5 This ethical standard is very easy to implement<br>☐ 4<br>☐ 3<br>☐ 2<br>☐ 1 This ethical standard is very hard to implement	☐ 5 This ethical standard greatly protects trial participants<br>☐ 4<br>☐ 3<br>☐ 2<br>☐ 1 This ethical standard minimally protects trial participants	☐ 5 I strongly agree with this ethical standard<br>☐ 4<br>☐ 3<br>☐ 2<br>☐ 1 I strongly disagree with this ethical standard
3. New prevention methods should be added to the prevention package based on consultation among all stakeholders.	☐ 5 I am very familiar with this ethical standard<br>☐ 4<br>☐ 3<br>☐ 2<br>☐ 1 I am not at all familiar with this ethical standard	☐ 5 This ethical standard is very easy to understand<br>☐ 4<br>☐ 3<br>☐ 2<br>☐ 1 This ethical standard is very hard to understand	☐ 5 This ethical standard is very easy to implement<br>☐ 4<br>☐ 3<br>☐ 2<br>☐ 1 This ethical standard is very hard to implement	☐ 5 This ethical standard greatly protects trial participants<br>☐ 4<br>☐ 3<br>☐ 2<br>☐ 1 This ethical standard minimally protects trial participants	☐ 5 I strongly agree with this ethical standard<br>☐ 4<br>☐ 3<br>☐ 2<br>☐ 1 I strongly disagree with this ethical standard
4. The protocol should describe how stakeholders will negotiate adding new methods to the risk reduction package.	☐ 5 I am very familiar with this ethical standard<br>☐ 4<br>☐ 3<br>☐ 2<br>☐ 1 I am not at all familiar with this ethical standard	☐ 5 This ethical standard is very easy to understand<br>☐ 4<br>☐ 3<br>☐ 2<br>☐ 1 This ethical standard is very hard to understand	☐ 5 This ethical standard is very easy to implement<br>☐ 4<br>☐ 3<br>☐ 2<br>☐ 1 This ethical standard is very hard to implement	☐ 5 This ethical standard greatly protects trial participants<br>☐ 4<br>☐ 3<br>☐ 2<br>☐ 1 This ethical standard minimally protects trial participants	☐ 5 I strongly agree with this ethical standard<br>☐ 4<br>☐ 3<br>☐ 2<br>☐ 1 I strongly disagree with this ethical standard
5. Trials should not be conducted when agreements have not been reached among all stakeholders regarding the standard of prevention.	☐ 5 I am very familiar with this ethical standard<br>☐ 4<br>☐ 3<br>☐ 2<br>☐ 1 I am not at all familiar with this ethical standard	☐ 5 This ethical standard is very easy to understand<br>☐ 4<br>☐ 3<br>☐ 2<br>☐ 1 This ethical standard is very hard to understand	☐ 5 This ethical standard is very easy to implement<br>☐ 4<br>☐ 3<br>☐ 2<br>☐ 1 This ethical standard is very hard to implement	☐ 5 This ethical standard greatly protects trial participants<br>☐ 4<br>☐ 3<br>☐ 2<br>☐ 1 This ethical standard minimally protects trial participants	☐ 5 I strongly agree with this ethical standard<br>☐ 4<br>☐ 3<br>☐ 2<br>☐ 1 I strongly disagree with this ethical standard
6. Communities should be meaningfully involved in determining the type, scope, and duration of HIV prevention services that will be available to participants and communities.	☐ 5 I am very familiar with this ethical standard<br>☐ 4<br>☐ 3<br>☐ 2<br>☐ 1 I am not at all familiar with this ethical standard	☐ 5 This ethical standard is very easy to understand<br>☐ 4<br>☐ 3<br>☐ 2<br>☐ 1 This ethical standard is very hard to understand	☐ 5 This ethical standard is very easy to implement<br>☐ 4<br>☐ 3<br>☐ 2<br>☐ 1 This ethical standard is very hard to implement	☐ 5 This ethical standard greatly protects trial participants<br>☐ 4<br>☐ 3<br>☐ 2<br>☐ 1 This ethical standard minimally protects trial participants	☐ 5 I strongly agree with this ethical standard<br>☐ 4<br>☐ 3<br>☐ 2<br>☐ 1 I strongly disagree with this ethical standard
7. The provision of risk reduction interventions should be monitored.	☐ 5 I am very familiar with this ethical standard<br>☐ 4<br>☐ 3<br>☐ 2<br>☐ 1 I am not at all familiar with this ethical standard	☐ 5 This ethical standard is very easy to understand<br>☐ 4<br>☐ 3<br>☐ 2<br>☐ 1 This ethical standard is very hard to understand	☐ 5 This ethical standard is very easy to implement<br>☐ 4<br>☐ 3<br>☐ 2<br>☐ 1 This ethical standard is very hard to implement	☐ 5 This ethical standard greatly protects trial participants<br>☐ 4<br>☐ 3<br>☐ 2<br>☐ 1 This ethical standard minimally protects trial participants	☐ 5 I strongly agree with this ethical standard<br>☐ 4<br>☐ 3<br>☐ 2<br>☐ 1 I strongly disagree with this ethical standard

8. Stakeholders should discuss disseminating results about how the standard of prevention was implemented in the trial.	☐ 5 I am very familiar with this ethical standard<br>☐ 4<br>☐ 3<br>☐ 2<br>☐ 1 I am not at all familiar with this ethical standard	☐ 5 This ethical standard is very easy to understand<br>☐ 4<br>☐ 3<br>☐ 2<br>☐ 1 This ethical standard is very hard to understand	☐ 5 This ethical standard is very easy to implement<br>☐ 4<br>☐ 3<br>☐ 2<br>☐ 1 This ethical standard is very hard to implement	☐ 5 This ethical standard greatly protects trial participants<br>☐ 4<br>☐ 3<br>☐ 2<br>☐ 1 This ethical standard minimally protects trial participants	☐ 5 I strongly agree with this ethical standard<br>☐ 4<br>☐ 3<br>☐ 2<br>☐ 1 I strongly disagree with this ethical standard
9. Researchers and trial sponsors should collaborate with government to strengthen capacity to deliver HIV prevention services.	☐ 5 I am very familiar with this ethical standard<br>☐ 4<br>☐ 3<br>☐ 2<br>☐ 1 I am not at all familiar with this ethical standard	☐ 5 This ethical standard is very easy to understand<br>☐ 4<br>☐ 3<br>☐ 2<br>☐ 1 This ethical standard is very hard to understand	☐ 5 This ethical standard is very easy to implement<br>☐ 4<br>☐ 3<br>☐ 2<br>☐ 1 This ethical standard is very hard to implement	☐ 5 This ethical standard greatly protects trial participants<br>☐ 4<br>☐ 3<br>☐ 2<br>☐ 1 This ethical standard minimally protects trial participants	☐ 5 I strongly agree with this ethical standard<br>☐ 4<br>☐ 3<br>☐ 2<br>☐ 1 I strongly disagree with this ethical standard
10. In the informed consent process, trial participants should be told what HIV prevention services they will receive.	☐ 5 I am very familiar with this ethical standard<br>☐ 4<br>☐ 3<br>☐ 2<br>☐ 1 I am not at all familiar with this ethical standard	☐ 5 This ethical standard is very easy to understand<br>☐ 4<br>☐ 3<br>☐ 2<br>☐ 1 This ethical standard is very hard to understand	☐ 5 This ethical standard is very easy to implement<br>☐ 4<br>☐ 3<br>☐ 2<br>☐ 1 This ethical standard is very hard to implement	☐ 5 This ethical standard greatly protects trial participants<br>☐ 4<br>☐ 3<br>☐ 2<br>☐ 1 This ethical standard minimally protects trial participants	☐ 5 I strongly agree with this ethical standard<br>☐ 4<br>☐ 3<br>☐ 2<br>☐ 1 I strongly disagree with this ethical standard
11. Trial participants should get access to optimal care and treatment for HIV infection, including ART.	☐ 5 I am very familiar with this ethical standard<br>☐ 4<br>☐ 3<br>☐ 2<br>☐ 1 I am not at all familiar with this ethical standard	☐ 5 This ethical standard is very easy to understand<br>☐ 4<br>☐ 3<br>☐ 2<br>☐ 1 This ethical standard is very hard to understand	☐ 5 This ethical standard is very easy to implement<br>☐ 4<br>☐ 3<br>☐ 2<br>☐ 1 This ethical standard is very hard to implement	☐ 5 This ethical standard greatly protects trial participants<br>☐ 4<br>☐ 3<br>☐ 2<br>☐ 1 This ethical standard minimally protects trial participants	☐ 5 I strongly agree with this ethical standard<br>☐ 4<br>☐ 3<br>☐ 2<br>☐ 1 I strongly disagree with this ethical standard
12. Trials should not be conducted when agreements have not been reached among all stakeholders regarding access to care and treatment.	☐ 5 I am very familiar with this ethical standard<br>☐ 4<br>☐ 3<br>☐ 2<br>☐ 1 I am not at all familiar with this ethical standard	☐ 5 This ethical standard is very easy to understand<br>☐ 4<br>☐ 3<br>☐ 2<br>☐ 1 This ethical standard is very hard to understand	☐ 5 This ethical standard is very easy to implement<br>☐ 4<br>☐ 3<br>☐ 2<br>☐ 1 This ethical standard is very hard to implement	☐ 5 This ethical standard greatly protects trial participants<br>☐ 4<br>☐ 3<br>☐ 2<br>☐ 1 This ethical standard minimally protects trial participants	☐ 5 I strongly agree with this ethical standard<br>☐ 4<br>☐ 3<br>☐ 2<br>☐ 1 I strongly disagree with this ethical standard
13. Communities should be meaningfully involved in determining the type, scope, and duration of treatment and care that will be available to participants and communities.	☐ 5 I am very familiar with this ethical standard<br>☐ 4<br>☐ 3<br>☐ 2<br>☐ 1 I am not at all familiar with this ethical standard	☐ 5 This ethical standard is very easy to understand<br>☐ 4<br>☐ 3<br>☐ 2<br>☐ 1 This ethical standard is very hard to understand	☐ 5 This ethical standard is very easy to implement<br>☐ 4<br>☐ 3<br>☐ 2<br>☐ 1 This ethical standard is very hard to implement	☐ 5 This ethical standard greatly protects trial participants<br>☐ 4<br>☐ 3<br>☐ 2<br>☐ 1 This ethical standard minimally protects trial participants	☐ 5 I strongly agree with this ethical standard<br>☐ 4<br>☐ 3<br>☐ 2<br>☐ 1 I strongly disagree with this ethical standard
14. Agreements on who will finance, deliver and monitor care and treatment should be documented.	☐ 5 I am very familiar with this ethical standard<br>☐ 4<br>☐ 3<br>☐ 2<br>☐ 1 I am not at all familiar with this ethical standard	☐ 5 This ethical standard is very easy to understand<br>☐ 4<br>☐ 3<br>☐ 2<br>☐ 1 This ethical standard is very hard to understand	☐ 5 This ethical standard is very easy to implement<br>☐ 4<br>☐ 3<br>☐ 2<br>☐ 1 This ethical standard is very hard to implement	☐ 5 This ethical standard greatly protects trial participants<br>☐ 4<br>☐ 3<br>☐ 2<br>☐ 1 This ethical standard minimally protects trial participants	☐ 5 I strongly agree with this ethical standard<br>☐ 4<br>☐ 3<br>☐ 2<br>☐ 1 I strongly disagree with this ethical standard

15. Stakeholders should discuss disseminating results about how access to care was implemented in the trial.	☐ 5<br>☐ 4<br>☐ 3<br>☐ 2<br>☐ 1<br>I am very familiar with this ethical standard<br>I am not at all familiar with this ethical standard	☐ 5<br>☐ 4<br>☐ 3<br>☐ 2<br>☐ 1<br>This ethical standard is very easy to understand<br>This ethical standard is very hard to understand	☐ 5<br>☐ 4<br>☐ 3<br>☐ 2<br>☐ 1<br>This ethical standard is very easy to implement<br>This ethical standard is very hard to implement	☐ 5<br>☐ 4<br>☐ 3<br>☐ 2<br>☐ 1<br>This ethical standard greatly protects trial participants<br>This ethical standard minimally protects trial participants	☐ 5<br>☐ 4<br>☐ 3<br>☐ 2<br>☐ 1<br>I strongly agree with this ethical standard<br>I strongly disagree with this ethical standard
16. Researchers and trials sponsors should collaborate with government to strengthen capacity to deliver care services.	☐ 5<br>☐ 4<br>☐ 3<br>☐ 2<br>☐ 1<br>I am very familiar with this ethical standard<br>I am not at all familiar with this ethical standard	☐ 5<br>☐ 4<br>☐ 3<br>☐ 2<br>☐ 1<br>This ethical standard is very easy to understand<br>This ethical standard is very hard to understand	☐ 5<br>☐ 4<br>☐ 3<br>☐ 2<br>☐ 1<br>This ethical standard is very easy to implement<br>This ethical standard is very hard to implement	☐ 5<br>☐ 4<br>☐ 3<br>☐ 2<br>☐ 1<br>This ethical standard greatly protects trial participants<br>This ethical standard minimally protects trial participants	☐ 5<br>☐ 4<br>☐ 3<br>☐ 2<br>☐ 1<br>I strongly agree with this ethical standard<br>I strongly disagree with this ethical standard
17. In the informed consent process, trial participants should be told what care and treatment services they will receive.	☐ 5<br>☐ 4<br>☐ 3<br>☐ 2<br>☐ 1<br>I am very familiar with this ethical standard<br>I am not at all familiar with this ethical standard	☐ 5<br>☐ 4<br>☐ 3<br>☐ 2<br>☐ 1<br>This ethical standard is very easy to understand<br>This ethical standard is very hard to understand	☐ 5<br>☐ 4<br>☐ 3<br>☐ 2<br>☐ 1<br>This ethical standard is very easy to implement<br>This ethical standard is very hard to implement	☐ 5<br>☐ 4<br>☐ 3<br>☐ 2<br>☐ 1<br>This ethical standard greatly protects trial participants<br>This ethical standard minimally protects trial participants	☐ 5<br>☐ 4<br>☐ 3<br>☐ 2<br>☐ 1<br>I strongly agree with this ethical standard<br>I strongly disagree with this ethical standard
18. Trials should only take place in communities where participants will have ongoing access to psychosocial services and legal support.	☐ 5<br>☐ 4<br>☐ 3<br>☐ 2<br>☐ 1<br>I am very familiar with this ethical standard<br>I am not at all familiar with this ethical standard	☐ 5<br>☐ 4<br>☐ 3<br>☐ 2<br>☐ 1<br>This ethical standard is very easy to understand<br>This ethical standard is very hard to understand	☐ 5<br>☐ 4<br>☐ 3<br>☐ 2<br>☐ 1<br>This ethical standard is very easy to implement<br>This ethical standard is very hard to implement	☐ 5<br>☐ 4<br>☐ 3<br>☐ 2<br>☐ 1<br>This ethical standard greatly protects trial participants<br>This ethical standard minimally protects trial participants	☐ 5<br>☐ 4<br>☐ 3<br>☐ 2<br>☐ 1<br>I strongly agree with this ethical standard<br>I strongly disagree with this ethical standard
19. Trials should help to develop HIV care services within the host country.	☐ 5<br>☐ 4<br>☐ 3<br>☐ 2<br>☐ 1<br>I am very familiar with this ethical standard<br>I am not at all familiar with this ethical standard	☐ 5<br>☐ 4<br>☐ 3<br>☐ 2<br>☐ 1<br>This ethical standard is very easy to understand<br>This ethical standard is very hard to understand	☐ 5<br>☐ 4<br>☐ 3<br>☐ 2<br>☐ 1<br>This ethical standard is very easy to implement<br>This ethical standard is very hard to implement	☐ 5<br>☐ 4<br>☐ 3<br>☐ 2<br>☐ 1<br>This ethical standard greatly protects trial participants<br>This ethical standard minimally protects trial participants	☐ 5<br>☐ 4<br>☐ 3<br>☐ 2<br>☐ 1<br>I strongly agree with this ethical standard<br>I strongly disagree with this ethical standard
20. Care approaches, and their successes and failures, should be carefully documented.	☐ 5<br>☐ 4<br>☐ 3<br>☐ 2<br>☐ 1<br>I am very familiar with this ethical standard<br>I am not at all familiar with this ethical standard	☐ 5<br>☐ 4<br>☐ 3<br>☐ 2<br>☐ 1<br>This ethical standard is very easy to understand<br>This ethical standard is very hard to understand	☐ 5<br>☐ 4<br>☐ 3<br>☐ 2<br>☐ 1<br>This ethical standard is very easy to implement<br>This ethical standard is very hard to implement	☐ 5<br>☐ 4<br>☐ 3<br>☐ 2<br>☐ 1<br>This ethical standard greatly protects trial participants<br>This ethical standard minimally protects trial participants	☐ 5<br>☐ 4<br>☐ 3<br>☐ 2<br>☐ 1<br>I strongly agree with this ethical standard<br>I strongly disagree with this ethical standard

21. Do you have any comments or thoughts about the ethical standards listed in this questionnaire?

22. To what extent do you think that HIV vaccine trial sites in South Africa are able to meet these standards?

23. Please indicate whether you think it is essential or not that South African HIV vaccine trial participants get access to the following preventative measures:

A. Male condoms	☐ Essential	☐ Nice-to-have but not essential
B. Female condoms	☐ Essential	☐ Nice-to-have but not essential
C. Risk reduction counselling	☐ Essential	☐ Nice-to-have but not essential
D. Sterile injecting equipment	☐ Essential	☐ Nice-to-have but not essential
E. Treatment of STIs	☐ Essential	☐ Nice-to-have but not essential
F. Post-exposure prophylaxis (PEP)	☐ Essential	☐ Nice-to-have but not essential
G. Male circumcision	☐ Essential	☐ Nice-to-have but not essential

If funds were limited, which THREE of the above prevention services would you prioritize (A, B, C, D, etc.)? ☐ ☐ ☐

Other preventative measures that you consider ESSENTIAL to provide to participants ____________________

Other preventative measures that you consider NICE-TO-HAVE BUT NOT ESSENTIAL ____________________

Preventative measures (including those listed above) that you think should NOT be provided ____________________

24. Please indicate whether you think it is essential or not that South African HIV vaccine trial participants get access to the following care services:

A. Positive prevention counselling	☐ Essential	☐ Nice-to-have but not essential
B. Social support	☐ Essential	☐ Nice-to-have but not essential
C. TB prevention and treatment	☐ Essential	☐ Nice-to-have but not essential
D. Opportunistic infection prevention and treatment	☐ Essential	☐ Nice-to-have but not essential
E. STI treatment	☐ Essential	☐ Nice-to-have but not essential
F. Nutrition	☐ Essential	☐ Nice-to-have but not essential
G. Testing of CD4 counts/ viral loads	☐ Essential	☐ Nice-to-have but not essential
H. Antiretroviral therapy (ART)	☐ Essential	☐ Nice-to-have but not essential
I. Palliative care	☐ Essential	☐ Nice-to-have but not essential
J. Home-based care	☐ Essential	☐ Nice-to-have but not essential
K. Reproductive health care for pregnancy and childbirth	☐ Essential	☐ Nice-to-have but not essential
L. Prevention of mother to child transmission (pMTCT)	☐ Essential	☐ Nice-to-have but not essential

If funds were limited, which FIVE of the above care services would you prioritize (A, B, C, D, etc.)? ☐ ☐ ☐ ☐ ☐

Other care measures that you consider ESSENTIAL to provide to participants ______________________________

Other care measures that you consider NICE-TO-HAVE BUT NOT ESSENTIAL ______________________________

Care measures (including those listed above) that you think should NOT be provided ______________________________

Thank you for your participation!
